# Zoledronic Acid Treatment for Diffuse Sclerosing Osteomyelitis of the Mandible: A Case Report and Literature Review of Antiresorptive Therapy

**DOI:** 10.7759/cureus.88963

**Published:** 2025-07-29

**Authors:** Teruyuki Niimi, Ryosuke Miwa, Ken Kitagawa, Nagato Natsume, Yuki Sakamoto

**Affiliations:** 1 Division of Research and Treatment for Oral and Maxillofacial Congenital Anomalies, School of Dentistry, Aichi Gakuin University, Nagoya, JPN; 2 Oral Surgery, Kansai Medical University Medical Center, Moriguchi, JPN

**Keywords:** antiresorptive therapy, diffuse sclerosing osteomyelitis, mandible, persistent mandibular pain, zoledronic acid

## Abstract

Diffuse sclerosing osteomyelitis (DSO) is a rare, chronic aseptic osteomyelitis of the mandible characterized by resistance to conventional therapies including nonsteroidal anti-inflammatory drugs (NSAIDs), antibiotics, corticosteroids, and surgery. Diagnosis is often delayed due to nonspecific symptoms and lack of typical infectious signs, sometimes resulting in misdiagnosis. We report a case of a 58-year-old male patient with persistent mandibular pain initially misdiagnosed as trigeminal neuralgia and treated with microvascular decompression surgery without improvement. Subsequent imaging revealed bone sclerosis and resorption consistent with DSO. IV administration of zoledronic acid resulted in rapid and sustained pain relief, improved mouth opening, and radiographic bone regeneration without adverse events. A review of 139 reported cases treated with antiresorptive agents (ARAs) including bisphosphonates (BPs) and denosumab (DMB) demonstrated a high remission rate (95%), though treatment protocols varied widely. BPs appear preferable to DMB due to longer duration of effect and lower risk of medication-related osteonecrosis of the jaw (MRONJ). This case and literature review underscore the efficacy and safety of ARAs in managing DSO but highlight the need for standardized treatment guidelines and further prospective studies to optimize therapy.

## Introduction

Diffuse sclerosing osteomyelitis (DSO) is a rare form of aseptic osteomyelitis characterized by resistance to a wide range of treatments, including nonsteroidal anti-inflammatory drugs (NSAIDs), antibiotics, hyperbaric oxygen therapy, corticosteroids, and surgical interventions. Although potential associations with chronic recurrent multifocal osteomyelitis (CRMO) and SAPHO syndrome (synovitis, acne, pustulosis, hyperostosis, and osteitis) have been proposed, the exact etiology and pathogenesis of DSO remain poorly understood [1,2].

DSO most commonly affects the mandible, where it presents unique clinical challenges. The disease often causes persistent mandibular pain and swelling, and radiographically, it manifests as a combination of bone sclerosis and osteolytic changes. The mandibular location makes treatment particularly difficult due to the complex anatomy and the potential for chronicity. Conventional therapies frequently fail to achieve lasting remission, necessitating consideration of alternative treatments.

In recent years, antiresorptive agents (ARAs) such as bisphosphonates (BPs) and denosumab (DMB) have been reported to show efficacy in managing DSO [3-5]. However, clear guidelines regarding indications, agent selection, dosage, and treatment regimens for ARA therapy have yet to be established.

Unlike bacterial osteomyelitis, DSO typically lacks overt signs of infection such as purulent discharge, which often complicates early diagnosis-especially when radiographic abnormalities are minimal. In this report, we present a case of DSO that was initially misdiagnosed as trigeminal neuralgia and treated with microvascular decompression surgery. The patient was subsequently referred to our department, where administration of zoledronic acid led to a favorable clinical outcome.

## Case presentation

A 58-year-old male patient was referred to the Oral Care Clinic of the Aichi Gakuin University Dental Hospital for evaluation of persistent mandibular pain.

Approximately three years prior to his initial visit, the patient had experienced pain in the left mandible and consulted his general dentist. He was diagnosed with apical periodontitis of the left mandibular first molar and pericoronitis of the left mandibular third molar. Hemi-section of tooth #36 and extraction of tooth #38 were performed. However, his symptoms did not improve, and he was referred to the oral and maxillofacial surgery department of a local hospital. A CT scan was performed, but no abnormalities were detected, and the cause of his symptoms remained unknown.

Despite treatment with NSAIDs prescribed by his general dentist, the pain persisted, and he was referred to the pain clinic of a university hospital two years prior. Medications such as pregabalin and tramadol-acetaminophen (Tramalset®) were prescribed without effect. He was then referred to the neurosurgery department of another hospital, where both cranial CT and MRI revealed no abnormalities. Subsequently, on his own initiative, he visited an orthopedic clinic due to associated neck pain. There, he received medication, physical therapy, and neck injections, but no significant improvement was observed.

One year and eight months prior to his initial visit to our clinic, the patient underwent microvascular decompression surgery after being diagnosed with trigeminal neuralgia at a neurosurgery department. However, his symptoms remained unchanged. He was then referred to our Oral Care Clinic by his general dentist.

At the time of the initial visit, the patient was 163 cm tall and weighed 65 kg, with good nutritional status. He had borderline diabetes mellitus, managed with diet alone. No facial asymmetry was noted. He exhibited mild trismus, with a maximum mouth opening of 30 mm. He reported severe pain extending from the left mandibular molar area to the mandibular ramus. While NSAIDs and tramadol offered partial relief, the pain would return within four hours, and he had difficulty sleeping at night due to the discomfort. Blood tests showed no evidence of inflammation, with normal levels of C-reactive protein (CRP) and white blood cell counts. 

**Figure 1:**
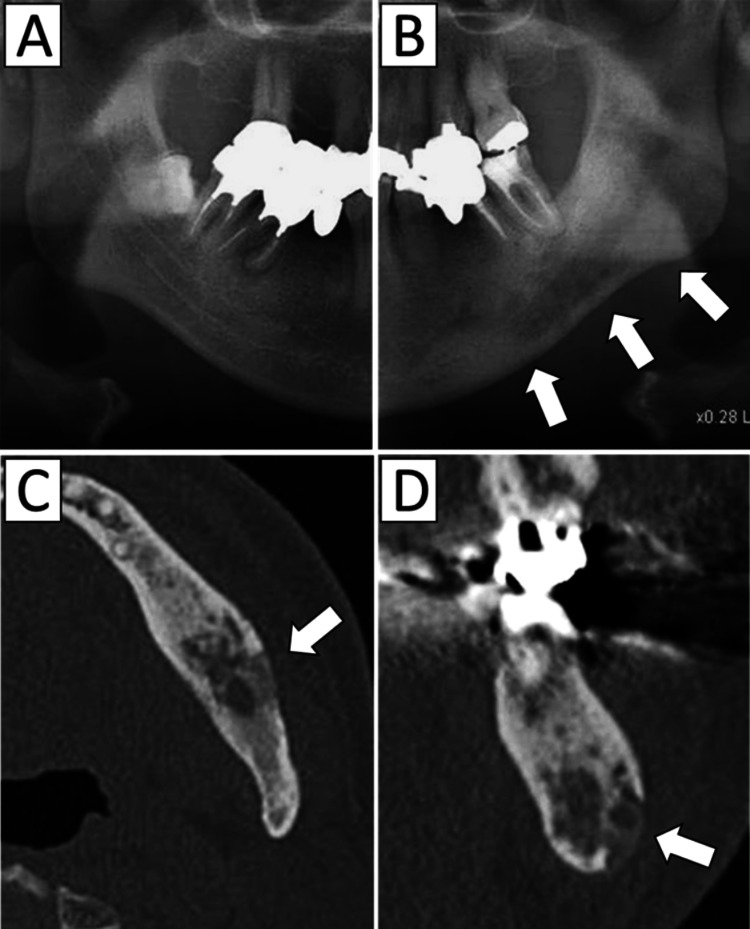
Imaging findings at the first visit to our department A: Panoramic radiograph of the unaffected side B: Panoramic radiograph of the affected side showing diffuse osteolytic changes (arrow) C, D: CT images demonstrating poorly defined osteolytic lesions (arrows) with surrounding osteosclerosis

**Figure 2:**
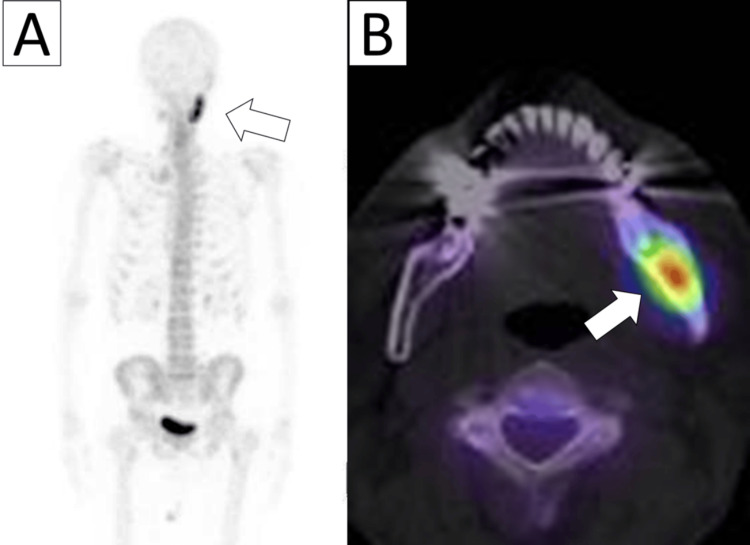
Tc-99m scintigraphy at the first visit to our department A: Increased tracer uptake is observed in the left mandible (arrow), with no abnormal findings in the rest of the skeletal system B: SPECT showing intense tracer accumulation in the left mandible (arrow) SPECT: Single Photon Emission CT

Panoramic radiography and CT imaging performed at our clinic revealed mixed areas of bone resorption and sclerosis extending from the left mandibular premolar region to the mandibular ramus (Figure 1). A Tc-99m bone scintigraphy scan showed intense uptake in the left mandible, with no abnormal accumulation in other regions (Figure 2). MRI was not performed at the initial presentation. Based on these findings, a diagnosis of DSO was made.

Although we administered antibiotics and NSAIDs, the relief was only temporary. Therefore, we proceeded with BP therapy. Since Aichi Gakuin University is a dental-only institution, treatment was carried out at Kansai Medical University Medical Center, a related medical facility, to allow appropriate management of potential adverse events. The patient received a single dose of 4 mg of zoledronic acid, diluted in 100 mL of normal saline and administered slowly by IV infusion over one hour. No adverse events occurred. The pain improved within a few days, although NSAIDs were still used as rescue medication.

**Figure 3:**
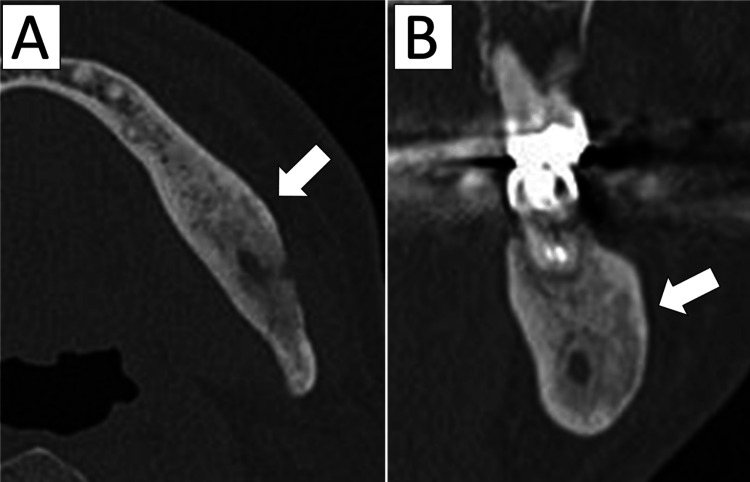
CT six months after BP therapy A, B: Although some radiolucent areas remain, ossification is observed in most of the previously osteolytic regions (arrows) BP: Bisphosphonate

The patient was later hospitalized at another facility for colorectal cancer surgery. Two months after completing cancer treatment, he returned to our clinic, reporting complete resolution of pain and discontinuation of NSAID use. CT imaging performed six months after zoledronic acid administration showed progressive bone formation in previously resorbed areas (Figure 3), and his trismus had also resolved. He has since remained under follow-up, and eight months post treatment, he reports no pain, with a favorable clinical course. Given that there have been reports of DSO recurring several years after BP treatment, we consider long-term follow-up to be necessary.

## Discussion

We report a case initially treated as trigeminal neuralgia but later diagnosed as DSO and successfully managed with zoledronic acid.

DSO is a noninfectious osteomyelitis occurring in the mandible that is resistant to antibiotics, NSAIDs, corticosteroids, and surgical treatments, representing an atypical form of osteomyelitis [6]. Unlike bacterial osteomyelitis, DSO rarely presents with fistula formation or purulent discharge, which often makes early diagnosis challenging. In reported cases, patients are frequently initially diagnosed with bacterial osteomyelitis and treated with antibiotics or surgery.

Nonbacterial inflammation, as seen in conditions like DSO, may result from impaired bone remodeling and vascular supply, leading to ischemia and increased intraosseous pressure. This, in turn, can stimulate nociceptors within the bone marrow and periosteum, resulting in chronic pain. Additionally, inflammatory cytokines released during abnormal bone turnover may further sensitize nerves and contribute to the pain experienced by patients [4].

We reviewed 139 reported cases of DSO treated with ARAs in the literature [7-24]. Cases associated with SAPHO syndrome or CRMO were excluded, and only those involving the mandible alone were included.

Among the cases, there were 34 males and 96 females (nine cases were unspecified), indicating a female predominance. The mean age was 36.9 years. The treatment regimens for ARAs in DSO have not been standardized and varied among reports. Regarding BPs, including overlapping uses, pamidronate was used in 72 cases, ibandronate in 27, zoledronate in 21, alendronate in 10, clodronate in seven, olpadronate in two, and risedronate in one case. DMB was used in two cases. Administration routes, dosages, and the number of administrations also varied widely. Most cases involved a single IV infusion, but pamidronate was sometimes given via consecutive infusions over three or five days. Oral administration was used with alendronate in eight cases and with risedronate in one case. One case combined alendronate with long-term tofacitinib therapy. The two cases treated with DMB received low-dose subcutaneous injections [7-24].

Regarding larger case series with more than 10 cases, Li et al. reported on 43 cases treated with pamidronate administered intravenously over three days [16]. Pain improved markedly in 40 cases, with 39 maintaining the effect for over six months. Maximum mouth opening improved from 28.5 mm to 38.1 mm, and radiographic improvement was observed in 74.4% of cases.

Van de Meent et al. described 18 cases: three achieved complete remission with more than two years of follow-up (mean 5.3 treatment cycles), three had complete remission but less than two years follow-up (mean 2 cycles), seven improved but occasionally used NSAIDs (mean 6.3 cycles), three were still undergoing treatment (mean 14.3 cycles), and two had unknown follow-up status [7].

Kusumoto et al. reported on 18 cases treated with IV zoledronic acid, all showing pain relief; however, eight cases required multiple doses [13]. The duration of effect was 80 months for single-dose cases and 32 months for those receiving multiple doses.

Frank et al. reported on 15 cases treated with ibandronate, all showing improved quality of life [17]. Otto et al. treated 11 cases with a single dose of ibandronate; 10 experienced near-complete pain relief, though four required retreatment.

Including smaller case series and single case reports, symptom remission was observed in 132 of 139 cases (95.0%). Although remission duration was not always clear, many reports indicated symptom resolution lasting several months or longer. However, many patients required retreatment: Kusumoto et al. reported eight of 18 cases, and Otto et al. reported four of 10 cases requiring repeated administration [10,13]. Due to limited sample sizes for each regimen, remission rates and duration of remission by treatment protocol could not be conclusively determined (Table 1).

**Table 1:** Literature review of reported cases PO: Orally; DMB: Denosumab; SC: Subcutaneous

Factors	Range (mean)/Number of patients
Age	5-81 (mean 36.9)
Sex	
Male	34
Female	96
Not described	9
Regimen (includes duplicates)	
Pamidronate×3 days (IV)	46
Ibandronate×1 day (IV)	27
Zoredronate×1 day (IV)	21
Pamidronate×5 days (IV)	16
Pamidronate×1 day (IV)	10
Alendronate once a week×1-36 months (PO)	9
Clodoronate×1 day (IV)	7
Olpadronate×3-5 days (IV/PO)	2
DMB×1 day (SC)	2
Alendronate×1 day (IV)	1
Risedronate×1 day (PO)	1
Treatment outcome	
Symptom relief or remission	132
No remarkable change	5
Unknown	2
Total	139

In the present case, at the patient’s first visit to his primary dentist approximately three years prior, no abnormalities were detected on panoramic radiography aside from apical periodontitis of the left mandibular first molar. Despite treatment of this tooth, the pain persisted, and the patient was referred to a pain clinic. Pharmacological therapy at the pain clinic was ineffective, and the patient was eventually diagnosed with trigeminal neuralgia and underwent microvascular decompression surgery at a neurosurgery department. However, during this period, imaging studies of the mandible such as CT and Tc-99m scintigraphy were not performed. Approximately two years after symptom onset, the patient visited the oral care clinic at Aichi Gakuin University Dental Hospital, where imaging examinations led to the diagnosis of DSO. Ultimately, zoledronic acid administration resulted in the long-lasting resolution of pain.

These findings highlight that early diagnosis of DSO can be difficult, and even when panoramic radiographs and CT scans show no abnormalities, suspicion of osteomyelitis warrants further evaluation by MRI or Tc-99m scintigraphy to assess for bone marrow inflammation.

Conventional treatments for bacterial osteomyelitis, including antibiotics and cortical bone resection, are generally ineffective for DSO. Montonen et al. first reported in 2001 the use of clodronate in six patients with DSO, showing significant pain reduction compared to placebo at six months [19]. In the same year, Soubrier et al. reported marked effectiveness of pamidronate in a DSO case [20]. Subsequent reports have documented the effectiveness of alendronate (2005); ibandronate (2015); zoledronate, risedronate, olpadronate (2022); and DMB (2023) [7,10,17,24].

Most cases treated with ARAs had previously received NSAIDs, surgery, or other therapies, yet persistent pain led to ARA administration. Early symptom resolution or improvement was reported in 95% of cases, with additional benefits including improvement of trismus and radiographic findings. Although adverse effects including influenza-like symptoms have been reported, no severe adverse events or medication-related osteonecrosis of the jaw (MRONJ) have been documented. These findings suggest that ARAs are a safe and effective treatment option for DSO.

However, the optimal choice of agent, dosage, and treatment regimen remain unclear, with various protocols reported. While symptom remission with DMB has been reported, BPs exhibit long-term bone deposition and prolonged antiresorptive effects, whereas DMB does not accumulate in bone and has a blood half-life of approximately one month. Consequently, DMB’s effect duration is limited and does not extend over years as with BPs. Furthermore, recent studies indicate a higher incidence of MRONJ with DMB than with BPs, with DMB-related MRONJ showing atypical CT findings and more difficult surgical management, as well as poorer clinical outcomes compared to BP-related MRONJ [25-27]. Therefore, we consider BPs to be superior to DMB as ARAs for treating DSO.

It is also important to note that the patient received BPs at a dose consistent with osteoporosis management, which is significantly lower than the dosage typically used in cancer patients with bone metastases. However, MRONJ can still occur in osteoporotic patients, albeit at a lower frequency. This suggests that other contributing factors, such as local anatomical vulnerability, chronic inflammation, or systemic conditions, may increase susceptibility even under low-dose therapy.

In our study including one case and 139 reported cases, no instances of MRONJ were observed. Although adverse events such as influenza-like symptoms may occur, no reports of serious adverse events following single or multiple BP administrations were found. These results indicate that BPs are safe and highly effective for treating DSO. Nonetheless, given the diversity of regimens and the limited number of cases, the optimal BP treatment protocol and dosing for DSO remain undetermined and warrant further investigation.

## Conclusions

DSO is a rare, noninfectious mandibular condition that is often resistant to conventional therapies. Our case highlights the potential of zoledronic acid, a BP, to provide lasting pain relief and radiographic improvement even after delayed diagnosis. A review of 139 cases supports the efficacy of ARAs, particularly BPs, in managing DSO, with high remission rates. However, standardized treatment protocols are lacking, and further prospective studies are needed to establish optimal regimens and assess long-term outcomes.
